# Development of a Robust Autofluorescence Lifetime Sensing Method for Use in an Endoscopic Application

**DOI:** 10.3390/s20071847

**Published:** 2020-03-26

**Authors:** Shuntaro Ito, Masaaki Hashimoto, Yoshihiro Taguchi

**Affiliations:** 1 School of Integrated Design Engineering, Keio University, 3-14-1, Hiyoshi, Yokohama 223-8522, Japan; ito@naga.sd.keio.ac.jp (S.I.); hashimoto@naga.sd.keio.ac.jp (M.H.); 2 Research Fellow of Japan Society for the Promotion of Science, 5-3-1 Kojimachi, Tokyo 102-0083, Japan; 3 Department of System Design Engineering, Keio University, 3-14-1, Hiyoshi, Yokohama 223-8522, Japan

**Keywords:** artifacts, autofluorescence lifetime, bio-mimicking phantom, endoscopic application, FAD, motion tracking, TCSPC

## Abstract

Endoscopic autofluorescence lifetime imaging is a promising technique for making quantitative and non-invasive diagnoses of abnormal tissue. However, motion artifacts caused by vibration in the direction perpendicular to the tissue surface in a body makes clinical diagnosis difficult. Thus, this paper proposes a robust autofluorescence lifetime sensing technique with a lens tracking system based on a laser beam spot analysis. Our optical setup can be easily mounted on the head of an endoscope. The variation in distance between the optical system and the target surface is tracked by the change in the spot size of the laser beam captured by the camera, and the lens actuator is feedback-controlled to suppress motion artifacts. The experimental results show that, when using a lens tracking system, the standard deviation of fluorescence lifetime is dramatically reduced. Furthermore, the validity of the proposed method is experimentally confirmed by using a bio-mimicking phantom that replicates the shape, optical parameters, and chemical component distribution of the cancerous tissue.

## 1. Introduction

Autofluorescence lifetime measurement has significant potential for providing quantitative diagnosis and analysis because autofluorescence lifetime, which is the time constant of autofluorescence intensity decay, facilitates the acquisition of biochemical information without the influence of optical conditions in sensing devices [1,2]. Autofluorescence lifetime has been used to measure the microenvironment state in a tissue and cells, such as the pH dependence, oxidation state, and fluorophore concentration changes. Islam et al. measured the pH dependence of the autofluorescence lifetime in a cell under a microscope [3]. Chacko et al. [4] and Skala et al. [5] investigated the cell metabolism using the autofluorescence lifetime. Autofluorescence lifetime sensing is one of the candidate diagnostic methods for optical biopsy; it has the advantage of non-invasive clinical diagnosis. Diagnosis using autofluorescence lifetime detected by an optical fiber probe has been widely investigated for many human organs, including the breast, stomach, skin, and prostate. Phipps et al. imaged resected specimens of human breast cancer using a fiber-based autofluorescence lifetime imaging device and showed that their system could distinguish cancerous tissue from fibrous tissue [6]. Munro et al. developed a flexible and endoscopic fluorescence lifetime imaging device and imaged a resected normal human stomach [7]. Moreover, autofluorescence lifetime imaging in vivo under restrained conditions has been performed using the contact type optical bundle probe [8,9,10,11,12,13]. For example, Sun et al. imaged brain tumors using an autofluorescence lifetime imaging device by contacting a probe to the cortex of a human brain undergoing craniotomy [10].

However, in non-contact endoscopic fluorescence lifetime measurements in digestive organs, organic motions induce strong motion artifacts distorting the fluorescence lifetime image, resulting in misdiagnosis. Motion artifacts arise from the contraction and dilatation of organs due to gastric motility, heart beating, breathing, and peristalsis. Several studies observed motion artifacts appearing in endoscopic fluorescence lifetime imaging [10,14,15]. Gorpas et al. showed motion artifacts being caused by in vitro vibrations of tissue by using an endoscopic fluorescence lifetime imaging device [14]. Furthermore, strong motion artifacts in the direction perpendicular to the human skin have been observed in fluorescence lifetime images by using a handheld device [15].

Vibration in a direction perpendicular to the tissue surface is a dominant source of noise in fluorescence lifetime imaging. Some studies have attempted to reduce motion artifacts by bringing the probe into contact with the sample [10,16] and detecting fluorophores at a high repetition rate [13,17]. However, these methods cannot be employed in endoscopic application because of deteriorating visibility and operability.

In this study, a robust fluorescence lifetime measurement system with the focus tracking method based on the laser spot imaging is proposed. In our previous study, a motion tracking method for a blood perfusion sensor had been developed. The proposed sensor was based on laser Doppler flowmetry with a confocal optical configuration [18]. The confocal signal is varied with the distance from the surface so that focus tracking can be achieved by monitoring the reflectance signal from the surface. Using this technique, robust blood perfusion sensing has been successfully conducted. In the present study, the body motion in the direction perpendicular to the surface is monitored by a camera that is compatible with the endoscope. The variation in the distance between the sample surface and the optical system is detected by the change in the spot size of the laser beam. The lens actuator is feedback-controlled by the results of the image analysis of the laser spot. In this case, using a high-quality laser source is not necessary for the confocal system. The validity of the proposed method is confirmed using a benchtop apparatus with a bio-mimicking phantom mounted on a single axial motor-driven stage imitating body motion.

## 2. Methods

**Figure 1:**
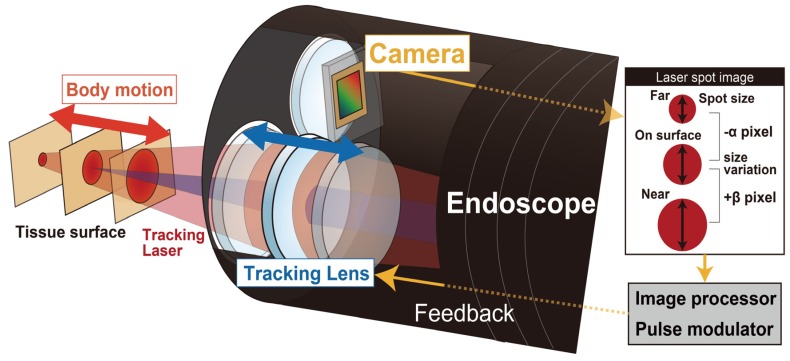
Principle of motion tracking system based on the feedback control of the lens actuator. The excitation laser is focused on the tissue surface, and the tracking laser is focused on the tissue. The spot size change of the tracking laser is captured by the camera, and the distance between the tissue and the endoscope is monitored via image analysis of the laser spot.

**Figure 2:**
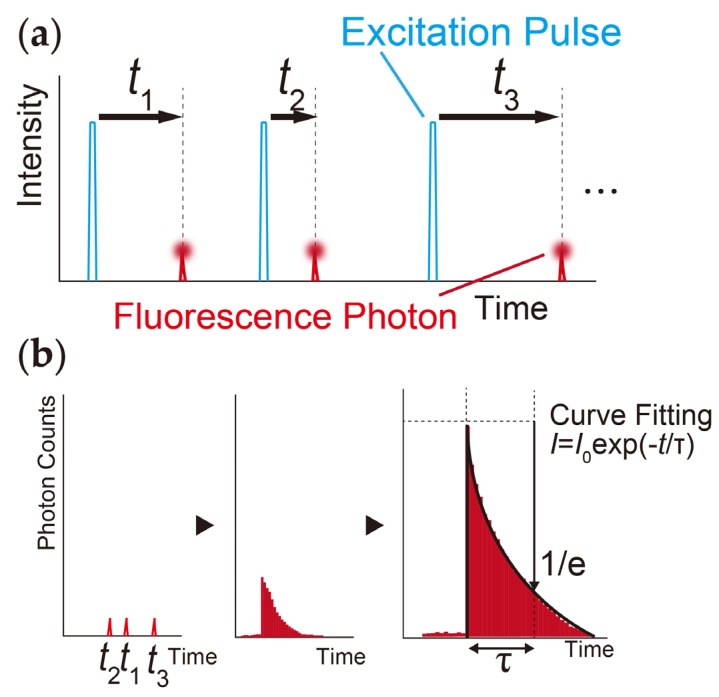
Principle of time correlated single photon (TCSPC). (**a**) A single photon emitted from the sample is detected, and the arrival time of each single photon after the excitation pulse is calculated. (**b**) A histogram of the arrival time of each photon is accumulated, and the autofluorescence signal decay is reconstructed using the histogram.

Figure 1 depicts the proposed idea of the motion tracking autofluorescence lifetime imaging system. In our method, fluorescence lifetime is measured using time correlated single photon counting (TCSPC) from the emitted fluorescence decay in the time domain. Figure 2 shows the working principle of TCSPC. Excited fluorescent photons from a high frequency pulse laser source are detected, and their arrival times are observed using the TCSPC module. A fluorescence decay signal is reconstructed from a histogram of the arrival times of the detected fluorescent single photons by using TCSPC. The fluorescent lifetime is analyzed via curve fitting using Equation (1).

(1) $$I_{t}=I_{0}\exp\left(-t/\tau \right)$$

where *I*_t_ is the fluorescence signal intensity, *I*_0_ is the initial fluorescence intensity, *t* is the time, and *τ* is the decay time constant (fluorescence lifetime). 

**Figure 3:**
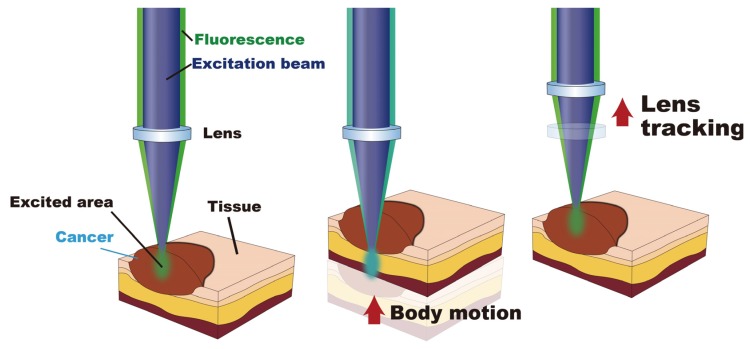
Motion artifact mechanism in autofluorescence lifetime sensing. The focal point of the excitation laser and the collection optical path vary due to body motion. Therefore, the undesired autofluorescence signal is mixed with the detected signal. By using lens tracking, the focal point is maintained in the appropriate position during body motion.

In fluorescence lifetime imaging with TCSPC, motions that are perpendicular to the tissue surface should be suppressed to achieve highly sensitive measurements of fluorescence lifetime. Figure 3 depicts a change in the excited area caused by tissue motion. A motion artifact appears in the fluorescence decay wave when the excited area exits the optimal measuring area due to body motion in the direction perpendicular to the tissue surface, resulting in changes in the detected photon counts and decay wave. Since both the targeted and undesired positions are excited during the measurement, a strong distortion of the fluorescence signal occurs, and the analyzed value of the fluorescence lifetime fluctuates. Also, the out of focus of the lens due to the body motion causes the degradation of the signal to noise ratio (S/N) of the detected photon signal, which affects the dispersion of analyzed data.

The detected signal is strongly affected by body motions such as heartbeats and gastro peristalsis. Therefore, a motion tracking method is necessary to maintain the distance between the target tissue and the optical system of the endoscope by controlling the lens actuator, as shown in Figure 1. In this system, the spot size change of the irradiating laser reflected from the tissue surface is utilized to control the lens actuator. The laser spot image is captured by the camera in the endoscope, and the diameter of the spot is analyzed in real-time by the image analysis program executed on the PC. 

The excitation laser for autofluorescence is focused on the tissue surface, and the focal position of the tracking laser is at a point far from that of the excitation laser. Hence, motion artifacts due to body motion can be compensated for by shifting the lens position synchronously using the feedback control of the actuator driver.

## 3. Experimental Setup

### 3.1. Motion Tracking Fluorescence Lifetime Imaging System

**Figure 4:**
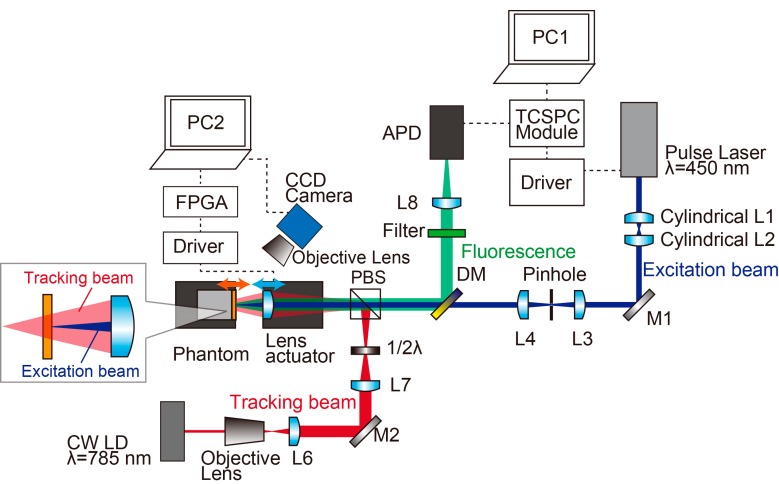
Experimental setup for the preliminary test of the proposed method. The pulse laser source (wavelength of 450 nm) is utilized as an excitation laser, and the continuous wave laser diode (CW LD, wavelength of 785 nm) is utilized as a tracking laser. Two beams are combined in the same optical path using the polarized beam splitter, and the autofluorescence from the sample is detected using avalanche photodiode (APD) through dichroic mirror (DM). The field-programmable gate array (FPGA) module can realize the high-speed feedback control of the lens actuator.

To verify the proposed motion tracking fluorescence lifetime imaging system, a bench-top apparatus that replicates the endoscopic configuration is constructed, as shown in Figure 4.

The experimental apparatus is composed of a fluorescence lifetime measurement system and a motion tracking system. The excitation light source of a pulsed laser head (LDH-D-C-450, PicoQuant, Berlin, Germany) emitted a 70 ps pulsed laser beam at λ = 450 nm, controlled by a driver (PDL-800-D, PicoQuant, Berlin, Germany) with a repetition rate of 5 MHz. To shape a pulsed laser beam suitable for sensing, two cylindrical lenses are used. Moreover, the Gaussian laser profile can be obtained using a spatial filter. The laser beam focused with a planoconvex lens excites the tissue surface, and the emitted photons are collected by the same lens. The fluorescence photons are detected by the avalanche photo diode (PD-1-C-T-C, MPD, Bolzano, Italy) with a 100 µm active sensing area on the detector through the short pass dichroic mirror (DMSP490, Thorlabs, NJ, USA), the band pass filter (FF02-525/40-25, Semrock, NY, USA), and a focusing lens. The TCSPC module (SPC-130EM, Becker&Hichkl, Berlin, Germany) counted a fluorescence photon each second and calculated the lifetime using the NIM signal from the laser driver. All these processes are controlled by a LabVIEW^TM^ program (National Instrument Inc., Austin, TX, USA) in a computer connected with the TCSPC module. 

The tracking laser (SWL-7513, Spectra-Physics, CA, USA) projects a laser spot onto the sample surface. The beam is expanded four times by the objective lens and the planoconvex lens to increase the beam diameter, and this high-NA condition gains tracking accuracy owing to the increase in the spot size variation during the movement of the tissue surface. The tracking laser beam is combined with the excitation laser beam by a polarized beam splitter (PBS121, Thorlabs, NJ, USA) and a half wave plate (AHWP05M-600, Thorlabs, NJ, USA). The CCD Camera (DFK21AU04, The Imaging Source, Bremen, Germany) captured the laser spot color images at 30 fps through the objective lens (SLMPLan 20x/0.35, Olympus, Tokyo, Japan) to analyze the spot diameter.

**Figure 5:**
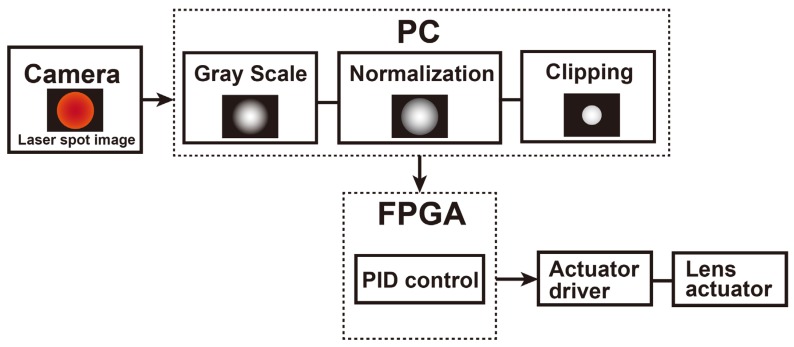
Signal processing flow for motion tracking using image analysis of the laser spot. A captured image of the laser spot by the camera is converted into a grayscale image followed by normalization and clipping based on a certain threshold value. After image analysis, the PID control is conducted by FPGA, and finally the lens actuator is feedback-controlled to compensate for the body motion.

A high-speed image analysis and low-delay feedback of the lens actuator is conducted using PC2 connected with the field-programmable gate array (FPGA) module (sbRIO-9637, National Instruments, Austin, TX, USA). Figure 5 depicts the analytical flow of processing the spot image and actuating the lens. First, the captured RGB image is converted into a grayscale image, followed by normalization to reduce the effect of the luminance change. Finally, the spot size is determined based on the threshold. The proportional-integral-derivative (PID) control signal is generated by the FPGA module by comparing the measured diameter of the laser spot and that of the set point. The objective lens is mounted on the ultrasonic linear actuator (XDT70-105, Techonohands Co. Ltd., Kanagawa, Japan) with the controller (TD-102, Technohands Co. Ltd., Kanagawa, Japan). The sample (bio-mimicking phantom) is mounted on the shaft motor (S120T, Nippon Pulse Motor, Tokyo, Japan) with the driver (MADHT1105L01, Panasonic, Osaka, Japan) to reproduce the body motion.

### 3.2. Bio-Mimicking Phantom 

**Figure 6:**
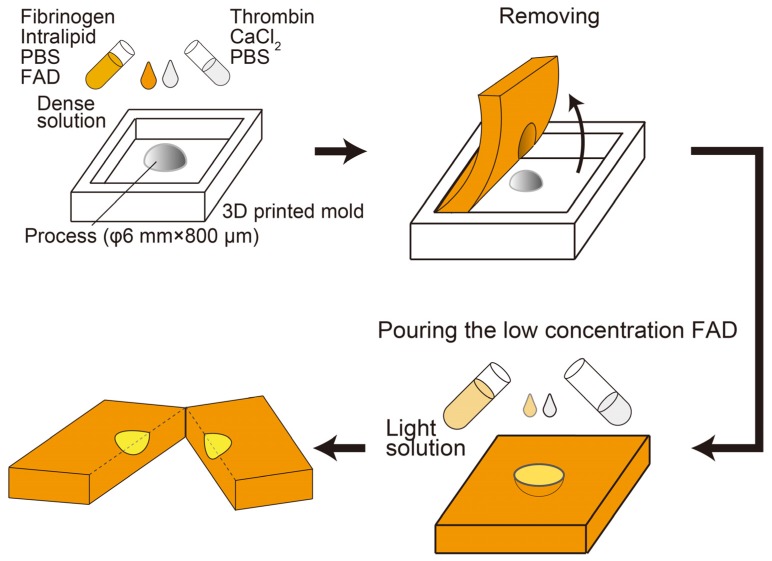
Fabrication process of flavin adenine dinucleotide (FAD) gel phantom to replicate the cancerous tissue. The process has two sequences: first, gelation of the high concentrated FAD sample in the 3D printed mold; second, pouring the low-concentration FAD solution into the cavity of the gel.

For the preliminary experiment to verify the proposed technique, a phantom mimicking the shape, optical characteristics, and flavin adenine dinucleotide (FAD) concentration distribution of the cancerous tissue [19] is prepared. FAD is a well-known autofluorescence chromophore, and the FAD autofluorescence can indicate the difference in metabolism between the normal and abnormal cells. The sample is a mixture of lipid emulsion, fibrinogen, thrombin, and FAD. The fabrication process of the bio-mimicking phantom is based on the fibrin clotting mechanism [20]. The proteolysis of fibrinogen by thrombin creates fibrin clots known as wound closure, and the optical scattering parameter is adjusted by adding the lipid emulsion. Figure 6 shows the process to make a solid gel phantom. In total, 0.16 mg of thrombin powder (T4648-1KU, Sigma-Aldrich, MO, USA) and 0.88 mg (8 µmole) of calcium chloride powder (036-00485, Wako pure chemical industries, Ltd., Osaka, Japan) are dissolved in 200 mL of saline (14249-95 D-PBS, Nacalai tesque, Kyoto, Japan). Moreover, 21 mg of fibrinogen powder (F8630-1G, Sigma-Aldrich, MO, USA) is dissolved in 300 mL of saline, to which 125 mL of 20% (w/v) Intralipid (I141-100ML, Sigma-Aldrich, MO, USA) is added. The concentration of Intralipid is determined by the concentration dependence of the optical attenuation coefficient [20], similar to that of the target tissue [21,22]. Two Fibrin/Intralipid/FAD solutions having different concentrations of FAD (0.1 wt% and 0.005 wt%) (16010-06, Nacalai Tesuque, Japan) resulting in differences of the autofluorescence lifetime are prepared to imitate the normal and cancerous tissue. A 3D printed mold is fabricated using UP Plus2 (Beijing Tiertime Technology Co. Ltd. Beijing, China). The projected mold pattern (height of 800 µm and diameter of φ6 mm) creates the recessed part in the fibrin matrix, and the lower FAD mixture is poured into the cavity to mimic the cancerous part. 

The fabricated bio-mimicking phantom is mounted on the shaft type linear motor and actuated sinusoidally (frequency: 0.25 Hz, displacement: 5 mm) and randomly (speed: 2 mm/s, displacement: 5 mm) to simulate the gastric and esophagus motility [23,24].

**Figure 7:**
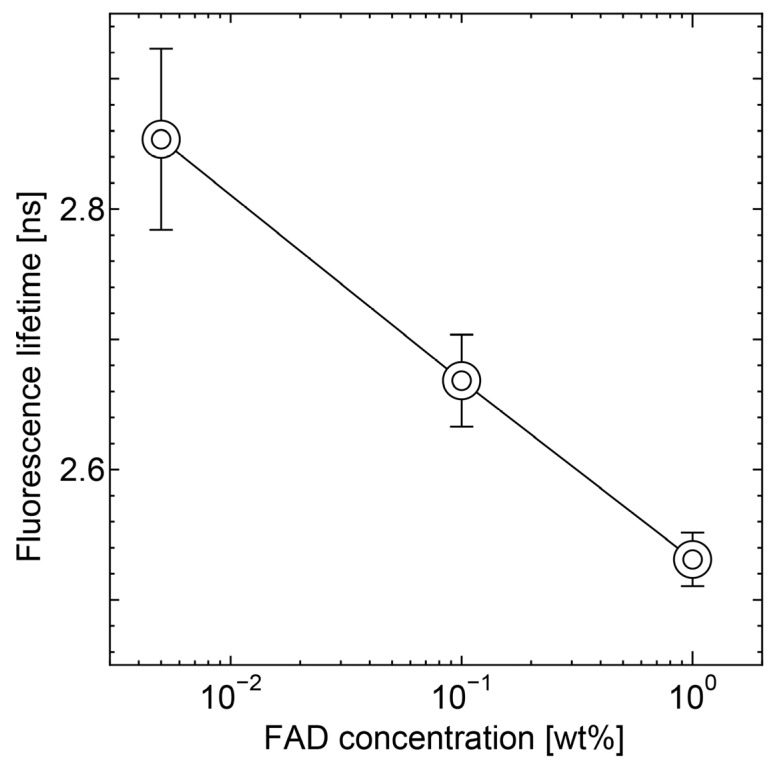
Validation of the experimental apparatus under the stationary state. The concentration dependence of FAD autofluorescence lifetime was successfully measured.

The concentration dependence of the fluorescence lifetime of the fibrin matrix was measured in the static condition. In Figure 7, the decreasing trend due to self-quenching was successfully observed. The detected value of the autofluorescence lifetime of FAD was within the range of literature values (2.7–2.9 ns) [3,25,26].

## 4. Results and Discussion

**Figure 8:**
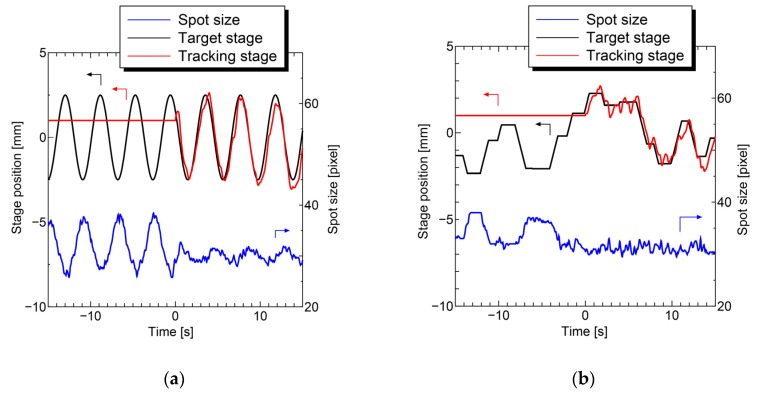
Motion tracking performance test under the vibration of the target with/without the control. (**a**) sinusoidal vibration and (**b**) random vibration.

First, the homogeneous FAD gel phantom was measured with and without motion tracking to verify the proposed system. The gel phantom (thickness of 2 mm) was mounted on the stage and actuated sinusoidally (Figure 8a) and randomly (Figure 8b). The lens actuator tracked the moving target smoothly, and the pixel size variation of the tracking laser beam was significantly suppressed in both cases. The precision of the focal length during motion tracking was estimated to be ± 0.6 mm based on the spot size variation.

**Figure 9:**
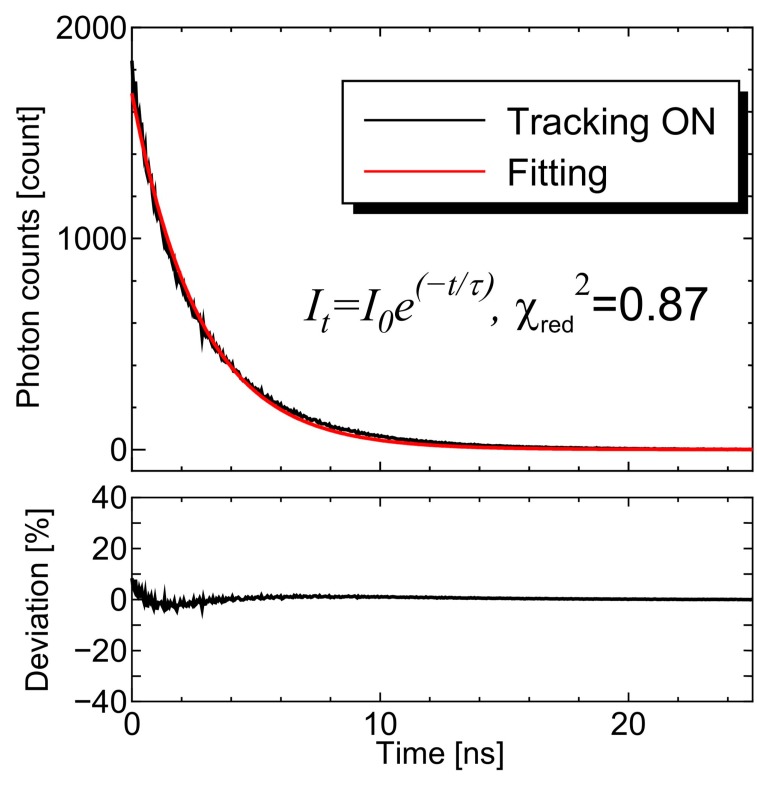
Detected autofluorescence decay signal with motion tracking under the sinusoidal vibration of the sample stage. The detected signal agreed well with the fitting curve given by Equation (1).

**Figure 10:**
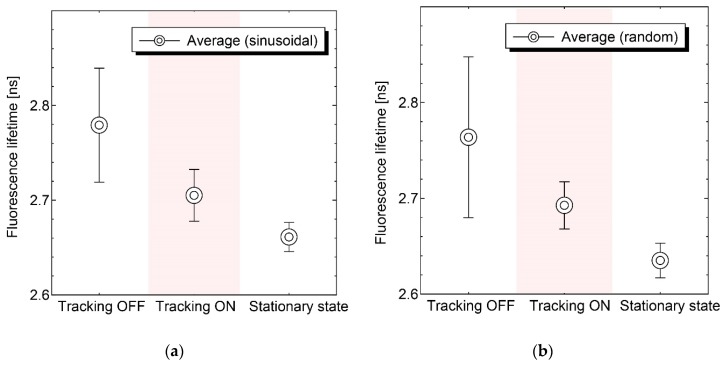
Comparison of detected autofluorescence lifetime under three conditions. In the case of the measurement without control, the deviation was significant, and the analyzed lifetime was significantly different from the stationary state because of the motion artifacts. (**a**) sinusoidal vibration and (**b**)random vibration.

Figure 9 presents the raw data of the autofluorescence decay curve that was reconstructed by the TCSPC module. A single exponential decay signal was successfully observed under the vibration condition (sinusoidal), and the decay curve agreed well with the fitting curve within ± 8% deviation. In this research, a single exponential decay fitting was utilized for the FAD phantom. The reduced chi-square value was estimated to be χ_red_^2^ = 0.87, therefore, the autofluorescence lifetime was adequately detected. As shown in Figure 10, the error due to the motion artifact was significantly suppressed with the proposed motion tracking system, and the measured fluorescence lifetime with the system approached the desired value calculated in the stationary state. The averaged value and the standard deviation are summarized in Table 1.

**Figure 11:**
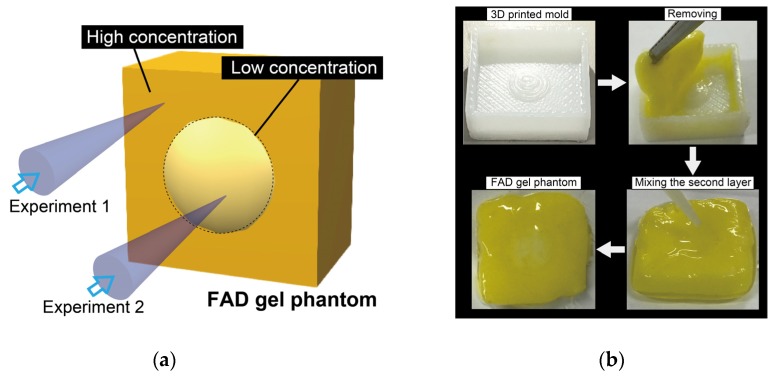
Bio-mimicking phantom of FAD. (**a**) Sensing point in the preliminary experiment and (**b**) fabricated bio-mimicking phantom.

**Figure 12:**
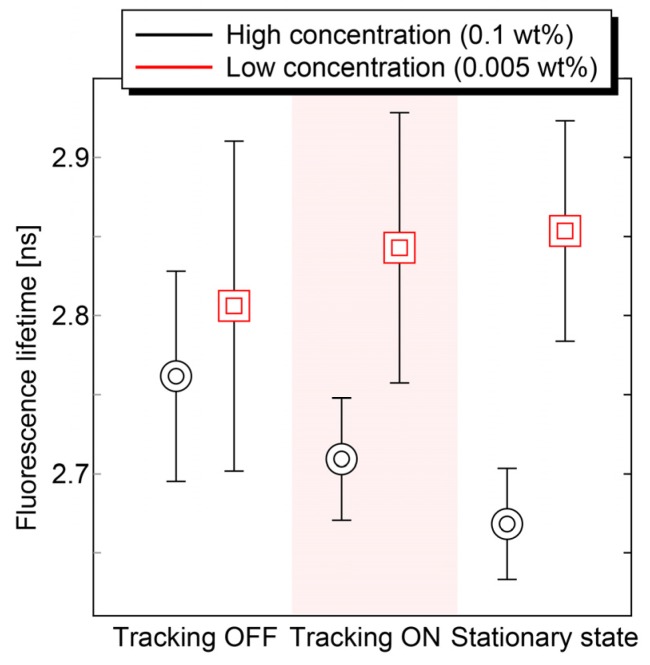
Comparison of detected autofluorescence lifetime and its standard deviation of bio-mimicking phantom with/without control. The concentration dependence of the autofluorescence of FAD was clearly observed with control. The fluctuation of detected autofluorescence lifetime caused by the motion artifacts was suppressed.

Finally, the bio-mimicking phantom with concentration distribution, as shown in Figure 11 was measured. Figure 12 shows the averaged fluorescence lifetime and its standard deviation of the central part (low concentration region) and its surrounding part (high concentration region) in the cases with/without motion tracking and the stationary state. In the case without motion tracking, it is difficult to distinguish the normal and abnormal part due to the strong fluctuation. On the other hand, the proposed motion tracking system enabled us to detect 0.13 ns changes of fluorescence lifetime with a standard deviation of ±40 ps, which is close to the value in the case of the stationary state. Consequently, the validity of our method was experimentally confirmed.

## 5. Conclusions

In conclusion, we proposed a robust autofluorescence lifetime sensing method for use in an endoscopic application. To suppress the motion artifacts arising in the endoscopic autofluorescence lifetime diagnosis, the motion tracking method was implemented in the autofluorescence lifetime measurement method. The sample vibration, which replicated the body motion, was monitored via image analysis of the irradiated laser spot, and feedback control of the lens actuator was successfully conducted. To validate the proposed method, a bio-mimicking phantom imitating the cancerous tissue was fabricated using a 3D-printed mold and FAD chromophore. The autofluorescence lifetimes of the normal and abnormal areas were distinguishable under the vibration condition using the motion tracking method. Thus, the robustness and applicability of the proposed method for the optical biopsy were experimentally confirmed.

## Figures and Tables

**Table 1:** Average and standard deviation of detected autofluorescence lifetime of flavin adenine dinucleotide (FAD) sample under two different motion artifacts (sinusoidal and random).

State	Motility	
	Sinusoidal	Random
Tracking OFF	2.78 ± 0.06 ns	2.76 ± 0.08 ns
Tracking ON	2.71 ± 0.03 ns	2.69 ± 0.02 ns
Stationary state	2.66 ± 0.02 ns	2.64 ± 0.02 ns
